# Progressive Seizure Aggravation in the Repeated 6-Hz Corneal Stimulation Model Is Accompanied by Marked Increase in Hippocampal p-ERK1/2 Immunoreactivity in Neurons

**DOI:** 10.3389/fncel.2016.00281

**Published:** 2016-12-16

**Authors:** Carmela Giordano, Anna M. Costa, Chiara Lucchi, Giuseppina Leo, Luc Brunel, Jean-Alain Fehrentz, Jean Martinez, Antonio Torsello, Giuseppe Biagini

**Affiliations:** ^1^Laboratory of Experimental Epileptology, Department of Biomedical, Metabolic and Neural Sciences, University of Modena and Reggio EmiliaModena, Italy; ^2^Department of Neurosciences, NOCSAE Hospital, AUSLModena, Italy; ^3^Max Mousseron Institute of Biomolecules, Centre National de la Recherche Scientifique (CNRS), University of Montpellier, École Nationale Supérieure de Chimie de Montpellier (ENSCM)Montpellier, France; ^4^Department of Medicine and Surgery, University of Milano-BicoccaMonza, Italy

**Keywords:** 6-Hz corneal stimulation, electrocorticography, epilepsy, EP-80317, extracellular signal-regulated kinase (ERK), FosB, hippocampus, growth hormone secretagogues

## Abstract

The 6-Hz corneal stimulation test is used to screen novel antiepileptic molecules to overcome the problem of drug refractoriness. Although recognized as a standard test, it has been evaluated only recently in the attempt to characterize the putative neuronal networks involved in seizures caused by corneal stimulation. In particular, by recording from the CA1 region we previously established that the hippocampus participates to propagation of seizure activity. However, these findings were not corroborated by using markers of neuronal activation such as FosB/ΔFosB antigens. In view of this discrepancy, we performed new experiments to characterize the changes in levels of phosphorylated extracellular signal-regulated kinases1/2 (p-ERK1/2), which are also used as markers of neuronal activation. To this aim, mice underwent corneal stimulation up to three different times, in three sessions separated by an interval of 3 days. To characterize a group in which seizures could be prevented by pharmacological treatment, we also considered pretreatment with the ghrelin receptor antagonist EP-80317 (330 μg/kg). Control mice were sham-treated. Video electrocorticographic (ECoG) recordings were obtained from mice belonging to each group of treatment. Animals were finally used to characterize the immunoreactivity for FosB/ΔFosB and p-ERK1/2 in the hippocampus. As previously shown, FosB/ΔFosB levels were highly increased throughout the hippocampus by the first induced seizure but, in spite of the progressively increased seizure severity, they were restored to control levels after the third stimulation. At variance, corneal stimulation caused a progressive increase in p-ERK1/2 immunoreactivity all over the hippocampus, especially in CA1, peaking in the third session. Predictably, EP-80317 administration reduced both duration and severity of seizures, prevented the increase in FosB/ΔFosB levels in the first session, and partially counteracted the increase in p-ERK1/2 levels in the third session. The vast majority of p-ERK1/2 immunopositive cells were co-labeled with FosB/ΔFosB antibodies, suggesting the existence of a relationship between the investigated markers in a subpopulation of neurons activated by seizures. These findings suggest that p-ERK1/2 are useful markers to define the aggravation of seizures and the response to anticonvulsant treatments. In particular, p-ERK1/2 expression clearly identified the involvement of hippocampal regions during seizure aggravation in the 6-Hz model.

## Introduction

In the early 1950s, the 6-Hz corneal stimulation model of “psychomotor seizures” was proposed as a tool to screen new anticonvulsants (Brown et al., 1953; White et al., 2002). More recently, it has been identified as a model for antiepileptic drug (AED)-resistant partial seizures (Barton et al., 2001; White et al., 2006). The reason for this was the unique result that led to identify the anticonvulsant effect of the clinically effective AED levetiracetam (Barton et al., 2001). Indeed, the 6-Hz corneal stimulation model has been included in the anticonvulsant screening project established by the National Institutes of Neurological Disorders and Stroke (Smith et al., 2007). Additionally, this model has been adopted to propose a new kindling protocol, also aimed at identifying new effective AEDs (Walrave et al., 2015). Despite the large use of 6-Hz corneal stimulation to induce convulsions, the cerebral regions participating to seizures induced in this model are still largely undetermined. Moreover, it is not established if this model may mimic at least some pathophysiological features of the most diffused type of refractory epilepsy, i.e., temporal lobe epilepsy (TLE; Curia et al., 2014). For instance, a prominent feature of TLE models is the presence of a process named “secondary epileptogenesis” (Williams et al., 2009; Pitkänen and Lukasiuk, 2011; Löscher et al., 2015; Van Nieuwenhuyse et al., 2015), by which a progressive aggravation of seizures occurs in epileptic animals.

Very few investigations attempted to define the neuronal networks involved in seizures induced by 6-Hz corneal stimulation (Barton et al., 2001; Duncan and Kohn, 2005; Giordano et al., 2015). By using c-Fos immunolocalization, different brain regions including the piriform cortex, cingulate and perirhinal cortices, neocortex and amygdala were found to present increased c-Fos levels immediately after seizure induction (Barton et al., 2001). Partially confirming these data, an autoradiographic analysis of ^14^C-2-deoxyglucose uptake revealed that neuronal cell populations were acutely involved by seizures in the neocortex, lateral amygdala and caudate-putamen (Duncan and Kohn, 2005). However, these experiments took into consideration only the responses to acute seizures, whereas the possibility that the identified cerebral regions could be involved in more durable phenomena, such as those characterizing secondary epileptogenesis (Pitkänen and Lukasiuk, 2011; Löscher et al., 2015), was never assessed. To this aim, we developed a model of repeated 6-Hz corneal stimulation in which an epileptogenic phenomenon, consisting of seizure worsening concomitant with progressive recruitment of the hippocampus, occurred (Giordano et al., 2015).

Although we previously demonstrated by electrocorticographic (ECoG) recordings that the CA1 region of the hippocampus was involved in seizure propagation when exposed to repeated 6-Hz corneal stimulation, this finding was not accompanied by durable changes in the expression of neuronal activity markers in the same region (Giordano et al., 2015). In particular, we evaluated the expression of FosB/ΔFosB antigens, which are known to be markedly induced either in seizure models (Chen et al., 1995; Mohapel et al., 2001), or in models of epilepsy (Morris et al., 2000; Biagini et al., 2005; Curia et al., 2013). Additionally, FosB/ΔFosB isoforms were directly involved in epileptogenesis since their deletion was associated with adult-onset spontaneous epilepsy (Yutsudo et al., 2013). Inconsistently, in our experiments we found that FosB/ΔFosB levels were initially elevated in the hippocampus, in response to the first corneally induced seizure, but then they went back to control levels by repeating the seizure induction. Moreover, the results obtained with FosB/ΔFosB were at odds with those observed by recording in CA1 of the same animals, in which we demonstrated the appearance of epileptic activity in association with aggravation of seizures (Giordano et al., 2015).

In order to further evaluate the involvement of hippocampal regions in mice stimulated by 6-Hz corneal electroshocks, we decided to characterize the expression of phosphorylated extracellular signal-regulated kinases 1/2 (p-ERK1/2) in response to repeated seizure induction. These markers have been found to be widely expressed in the hippocampus of animals exposed to seizures induced by kainate (Kim et al., 1994; Crespo-Biel et al., 2007), pilocarpine (Garrido et al., 1998; Berkeley et al., 2002; Houser et al., 2008), bicuculline (Gass et al., 1993; Ben et al., 2014), and dopamine D_1_ receptor agonists (Bozzi et al., 2011; Gangarossa et al., 2011, 2014; Bozzi and Borrelli, 2013). We also found p-ERK1/2 to be induced by audiogenic seizures in mice affected by fragile X syndrome (Curia et al., 2013) and, interestingly, changes in p-ERK1/2 have been associated to a different sensitivity to proconvulsant stimuli (Glazova et al., 2015). In order to characterize p-ERK1/2 expression in the repeated 6-Hz corneal stimulation model, we designed an experiment in which p-ERK1/2 was characterized in the hippocampus of mice exposed to one or three sequential seizures. Additionally, we considered the possibility to antagonize seizures by administering the anticonvulsant EP-80317 (Biagini et al., 2011), so to determine the modulation of neuronal activity markers to this pharmacological challenge. Overall, we identified p-ERK1/2 as a critical mediator involved in the hippocampal response to 6-Hz corneally induced seizures.

## Materials and Methods

### Animals and Treatments

Four-week-old male CD-1 mice (*n* = 44; Charles River, Calco, Italy) were used for this study. Six of these animals (control group) were used to determine basal levels of the investigated markers and were not treated or stimulated, but they were handled and exposed to the same procedure as the others. Nineteen were intraperitoneally (i.p.) saline-treated mice, and other 19 mice received i.p. injections of the ghrelin receptor antagonist EP-80317 (Haic-D-Mrp-D-Lys-Trp-D-Phe-Lys-NH_2_, produced by conventional solid phase synthesis; 330 μg/kg), previously shown to be an anticonvulsant (Biagini et al., 2011). Injections of EP-80317 or saline were performed 15 min before each session of corneal stimulation. All experiments were approved by the Italian Ministry of Health (Ministero della Salute) (92/2013) and performed in accordance with the European Directive 2010/63/EU.

### Corneal Stimulation Protocol

Mice were stimulated once and let to recover for 3 days before being stimulated again. Corneal stimulation was performed as previously described (Giordano et al., 2015). Briefly, a topical eye anesthetic (0.4% oxybuprocaine hydrochloride eye drops, Novesin, Novartis, Switzerland) was applied 10 min before stimulation. Stimulation (fixed current intensity of 32 mA, pulse width of 0.2 ms, duration of 3 s, frequency of 6 Hz) was delivered via corneal electrodes connected to a stimulator (ECT Unit 5780; Ugo Basile, Comerio, Italy). Seizure severity was ranked according to the following scores: 1, stunned posture and eye blinking; 2, head nodding, Straub tail and repetitive rhythmic movements (stereotypies) such as chewing; 3, unilateral or alternating forelimb clonus; 4, generalized tonic-clonic convulsions without loss of posture, or rearings; and 5, generalized tonic-clonic convulsions with loss of posture. Seizures were first scored immediately after corneal stimulation by direct observation, then reanalyzed on video recordings to quantify the duration of behavioral changes by an investigator unaware of the stimulation session. Recovery from seizures was defined by the reappearance of a normal exploratory behavior.

### Electrodes Implantation for Electrocorticographic (ECoG) Recordings

Few mice (*n* = 3, respectively, for mice treated with saline or EP-80317) were implanted to perform ECoG recording of the induced seizures. For electrode implantation, mice were anesthetized with ketamine (150 μg/g, i.p.) + xylazine (10 μg/g, i.p.). Guiding holes were drilled and epidural electrodes (stainless steel Ø = 1 mm; PlasticsOne, Roanoke, VA, USA) were implanted in frontal (Bregma 0 mm, 3 mm lateral from midline) and occipital cortices (Bregma −3.5 mm, 3 mm lateral from midline) of right hemisphere (Giordano et al., 2015). One electrode was implanted below lambda on the midline and used as reference. Electrodes were connected by steel wire to terminal gold pins (Bilaney Consultant GmbH, Düsseldorf, Germany) that were inserted in a plastic pedestal (PlasticsOne, Roanoke, VA, USA) cemented on the mouse head. At the end of the surgery, a gel containing 2.5 g lidocaine chloride, 0.5 g neomycin sulfate and 0.025 g fluocinolone acetonide (Neuflan gel; Molteni Farmaceutici, Scandicci FI, Italy) was applied to avoid sufferance and risk of infection. These mice were not used for immunohistochemistry to avoid any possible interference with expression of the investigated markers of stress cause by the implant.

### Video ECoG Recordings

Mice were placed in cages without cover to allow cable connection between headset and preamplifiers. Electrical brain activity was digitally filtered (0.3 Hz high-pass, 500 Hz low-pass), acquired at 1 kHz per channel, and stored on personal computer as the mathematical subtraction of traces of recording electrodes minus traces of reference electrode (only for epidural electrodes), using a PowerLab8/30 amplifier connected to 4 BioAmp preamplifiers (ADInstruments; Dunedin, Otago, New Zealand). Videos were digitally captured by a camera connected to the computer, which was synchronized to the EcoG traces by LabChart 7 Pro internal trigger.

### Immunohistochemistry

Mice (*n* = 6 controls; *n* = 16 for each treatment group) were deeply anesthetized with isoflurane (~10 s), 14–17 h after the seizure test. They were perfused transcardially with phosphate buffered saline (PBS, pH 7.4), followed by Zamboni’s fixative (pH 6.9). Brains were isolated and postfixed at 4°C in the same fixative for 24 h and then transferred into 15% and 30% sucrose solution (Vinet et al., 2016). Horizontal sections of 50 μm were cut using a freezing sliding microtome (Leica SM2000 R; Leica, Nussloch, Germany). Sections were washed in PBS, treated with 3% H_2_O_2_ in PBS (20 min) in order to quench endogenous peroxidase activity and washed again in PBS. Then, they were blocked 1 h in PBS/0.1% Triton X-100 containing 5% normal goat serum and incubated at 4°C with a rabbit polyclonal anti-FosB/ΔFosB (H-75, sc-7203, Santa Cruz Biotechnology, Santa Cruz, CA, USA; 1:250, overnight) and a mouse monoclonal anti-p-ERK1/2 (Thr202/Tyr204, cat-9106, Cell Signal Technology, Beverly, MA, USA; dilution 1:10,000, 48 h). After washing, sections were incubated for 1 h firstly with a biotinylated anti-rabbit or, respectively, anti-mouse secondary antibody (Vector Laboratories, Burlingame, CA, USA; 1:200) and secondly with the avidin-biotin-peroxidase complex (Elite ABC Kit; Vector Laboratories, Burlingame, CA, USA). The immunostaining was developed in 0.05% 3,3-diaminobenzidine tetrahydrochloride for 5 min (Sigma-Aldrich, Milan, Italy) by adding 0.03% H_2_O_2_.

### Image Analysis

Immunostained sections mounted on gelatin-coated slides and relative to bregma from −8.04 mm to −5.04 mm for hippocampal CA1, CA3, and dentate gyrus (DG) regions were analyzed using an Axioskop microscope (Carl Zeiss Vision GmbH, Munchen, Germany) equipped with a 10× objective. For each area of interest, images were digitally captured by a Sony CCD-IRIS B–W video camera, along the ventrodorsal direction of the brain (approximately 6–7 serial horizontal sections separated by 0.5 mm). A mouse brain atlas-C57BL/6J horizontal^1^ was used to assess brain sections. The number of immunoreactive cells per mm^2^ was quantified using the image analysis software KS300 (Carl Zeiss Vision GmbH), as previously detailed (Biagini et al., 2005; Curia et al., 2013; Giordano et al., 2015). The border of all analyzed regions was manually traced and the sampled areas, expressed in mm^2^, were automatically determined. Background values in each section were acquired from areas that did not contain any stained cell, such as the angular bundle. FosB/ΔFosB immunopositive cells were determined in each area as the number of immunopositive profiles after transformation in D-circles (i.e., the diameter of circles having the same area as measured) by considering a minimum cut-off value of 7 μm; p-ERK1/2 immunopositive cells were instead manually counted. Cell count was then normalized by the sampled area and expressed as cellular density (n/mm^2^). All measurements were taken bilaterally and the final values represent the left-right average.

### Double Immunofluorescence

Sections were washed thrice in PBS at room temperature and permeabilized for 1 h in PBS/0.1% Triton X-100 containing 5% normal goat serum. For double immunolabeling, sections were incubated overnight with rabbit polyclonal anti-FosB/ΔFosB (1:250) and mouse monoclonal anti-p-ERK1/2 (1:500) antibodies. After washing, sections were incubated for 3 h at room temperature with secondary antibodies Alexa Fluor 488-labeled goat anti-rabbit (A-11001; Invitrogen, Carlsbad, CA, USA; 1:500) and Alexa Fluor 594-labeled goat anti-mouse antibody (A-11005; Invitrogen, Carlsbad, CA, USA; 1:500). Sections were mounted and coverslipped with Mowiol. Images were acquired using a Leica TCS SP2 (Leica Laser technik, Heidelberg, Germany) confocal multiband scanning laser equipment with AOBS system adapted to an inverted Leica DM IRE2 microscope interfaced with an Argon-Kripton laser set at a power of 8 mW in each line (488, 568) and operating in the single scan acquisition mode. To minimize the noise and to keep a low photobleaching rate, we selected an acquisition time of 2 s per scan and averaged two scans to produce each 1024 × 1024 pixel image. All images of fluorescent neurons in CA1 were taken at HCX PL APO 40× magnification and the final images were the merge of a rostro-caudal series of 4–6 images in a depth of 4–6 μm**.** The number of double-labeled cells were counted using Fiji software^2^.

### Statistics

Seizure duration was analyzed using one-way analysis of variance (ANOVA) and the *post hoc* Fisher’s least significant difference (LSD) test. FosB/ΔFosB and p-ERK1/2 data were compared by using the same statistical approach. The Fisher’s exact test was used to compare scores of motor responses, classified as convulsions with or without loss of posture, in the various groups of treatment. All statistical analyses were performed using Sigmaplot 11 (Systat Software, San Jose, CA, USA). When not otherwise indicated, results are shown as mean ± standard error of the mean and *P* < 0.05 was considered statistically significant.

## Results

### Seizures Induced by Repeated 6-Hz Corneal Stimulation Were Attenuated by Treatment With EP-80317

**Figure 1:**
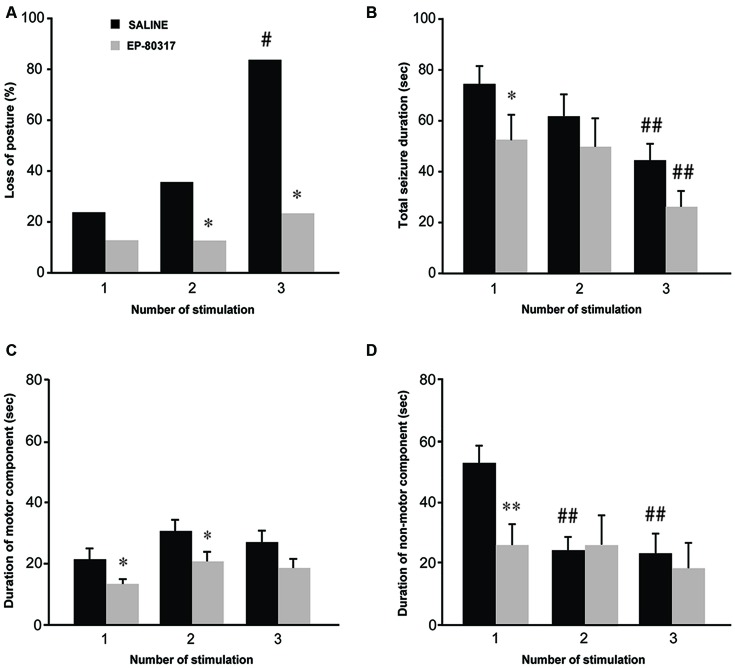
**Behavioral response to repeated 6-Hz corneal stimulation in mice treated with saline or EP-80317.** Seizures were evaluated for severity and duration. Severity was measured as the percentage of mice developing generalized tonic-clonic seizures associated with loss of posture **(A)**. Note that loss of posture occurred in a progressively higher percentage of saline-treated mice (^#^*P* < 0.05 vs. session 1 in the same treatment group, Fisher’s exact test; *P < 0.05 vs. saline-treated mice in sessions 2 and 3). The seizure duration **(B–D)** was evaluated separately for convulsions (**C**, motor component) and stunned posture (**D**, non-motor component), or as overall duration (**B**, including both components). Note that the overall duration decreased progressively after each session in both groups (^##^*P* < 0.01, session 3 vs. 1; Fisher’s least significant difference (LSD) test). Interestingly, a significantly lower overall duration was initially found in mice treated with EP-80317 (*P < 0.05 vs. saline-treated mice in session 1; Fisher’s LSD test). When considering only the motor component **(C)**, this was reduced by EP-80317 in the first two sessions (*P < 0.05). Concerning the non-motor component **(D)**, a significant decrease was found in saline-treated mice (^##^*P* < 0.01, session 2 and 3 vs. session 1). In mice treated with EP-80317 group, the non-motor component did not change in the various sessions and it was initially shorter than in saline-treated mice (**P < 0.01, session 1).

We analyzed severity and duration of seizures induced by 6-Hz corneal stimulation (Figure 1). Increased severity was determined as the development of generalized tonic-clonic convulsions with loss of posture, whereas seizure termination was established as the reappearance of a normal exploratory behavior. Consistent with previous findings (Giordano et al., 2015), the percentage of mice which displayed generalized tonic-clonic seizures associated with loss of posture significantly increased (*P* < 0.05, session 3 vs. session 1; Fisher’s exact test) in mice treated with saline (Figure 1A). Interestingly, only a limited number of mice treated with EP-80317 developed generalized tonic-clonic convulsions, being significantly different from saline-treated mice (*P* < 0.05, in both session 2 and session 3). Thus, by limiting generalized tonic-clonic seizures EP-80317 produced anticonvulsant effects which did not subside in the course of three different sessions of corneal stimulation.

The overall seizure duration was shorter in mice treated with EP-80317 compared to saline-treated mice (*P* < 0.05, Fisher’s LSD test) during the first session of seizure induction (Figure 1B). However, as mice more quickly recovered from seizures after the first induction (Giordano et al., 2015), the difference in the overall seizure duration was no more evident in the second and third session of corneal stimulation. Especially in the third session, the reduction in seizure duration was significant in both groups of treatment compared with the respective seizure duration of the first session (*P* < 0.01 for both groups, session 3 vs. session 1).

By further analyzing 6-Hz corneally induced seizures to distinguish motor (convulsions) from non-motor (stunned posture; see Giordano et al., 2015) components, we could explain the reason for the decreased seizure duration, which was due to reduction in the immobility period that followed convulsions. Precisely, the seizure motor component did not change its duration in saline-treated mice across the three different sessions of stimulation (Figure 1C), whereas the non-motor component was significantly shortened both in the second and third session of seizure induction (*P* < 0.01, session 2 and 3 vs. session 1 in saline-treated mice; Fisher’s LSD test; Figure 1D). The non-motor component was also significantly shorter in EP-80317 compared to saline-treated mice in the course of the first session of seizure induction (*P* < 0.01), but this difference was not found in the following sessions of corneal stimulation (Figure 1D). Notably, EP-80317 significantly shortened the motor component with respect to saline-treated mice in both the first and second session of corneal stimulation (*P* < 0.05, Fisher’s LSD test). However, in the third session both groups presented similar values, suggesting that a certain degree of resistance to the anticonvulsant effects of EP-80317 was developed by repeating the seizure induction.

**Figure 2:**
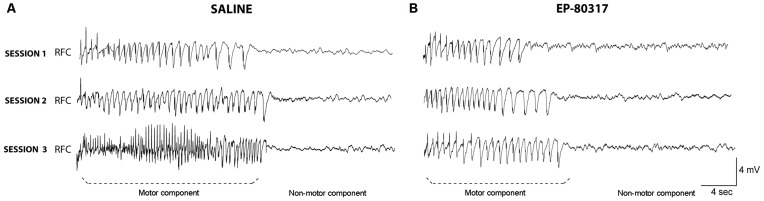
**Electrocorticographic (ECoG) activity during seizures induced by 6-Hz corneal stimulation.** EcoG traces obtained from frontal cortex of one out of three representative mice. Note that the trace clearly shows two components: (i) the ictal event corresponding to the motor component of the seizure; and (ii) the post-ictal event, characterized by flattening of basal activity corresponding to the non-motor component of seizures. Traces were respectively taken from a saline-treated mice **(A)** or an animal treated with EP-80317 **(B)**. No quantification is provided because of the low number of animals per group (*n* = 3). RFC, right frontal cortex.

In few mice we also performed ECoG recordings to compare the observed response with our previous results (Giordano et al., 2015). In these animals we confirmed that the electrographic seizure corresponded to the visually scored motor component quantified in Figure 1C, and that the non-motor component was represented by flattening of the electrographic signal, as indicated in Figure 2. However, the very low number of animals (*n* = 3 for each group) used for ECoG recordings did not allow any reliable quantitative analysis of the seizure characteristics.

### EP-80317 Prevented the Increase in FosB/ΔFosB Expression Initially Observed in the Hippocampus of Saline-Treated Mice

**Figure 3:**
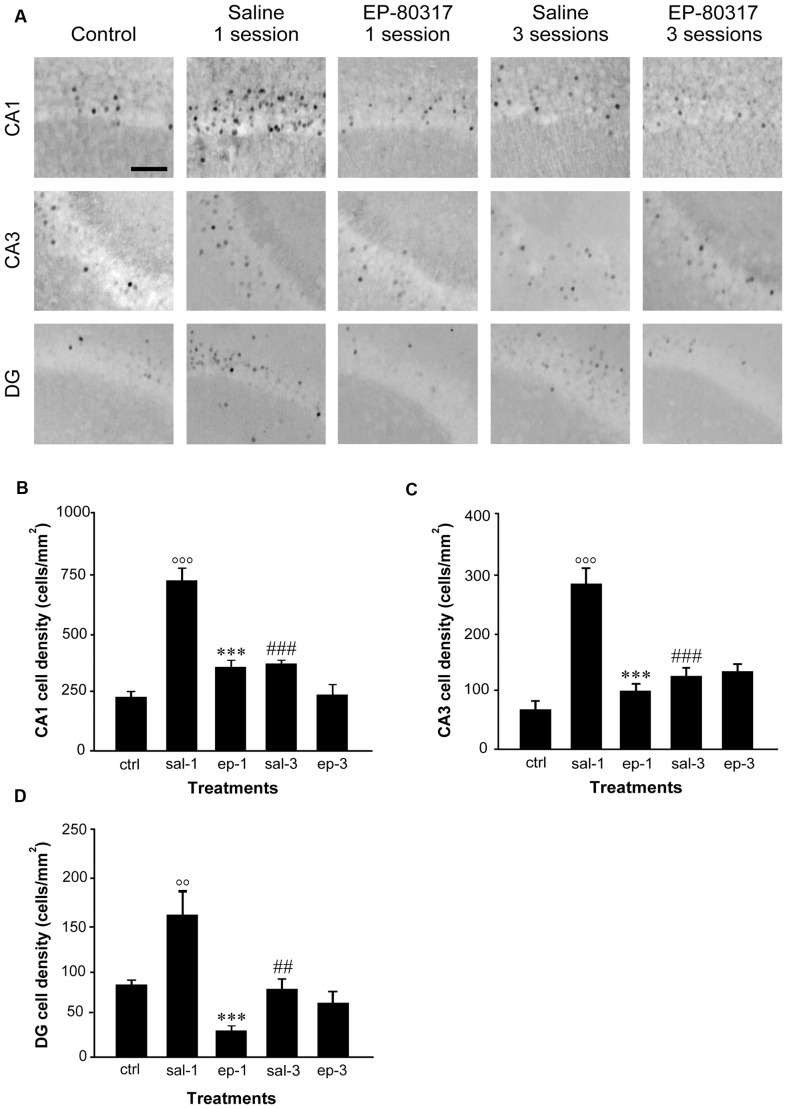
**FosB/ΔFosB immunoreactivity in hippocampal regions of mice treated with saline or EP-80317 and exposed to different sessions of 6-Hz corneal stimulation.** In **(A)**, FosB/ΔFosB immunoreactivity is illustrated in CA1, CA3, and dentate gyrus (DG), in a representative unstimulated control (ctrl) mouse. Besides, FosB/ΔFosB immunoreactivity is also shown in mice exposed to one or three different sessions of 6-Hz corneal stimulation, and pretreated with saline (sal) or EP-80317 (ep). Immunoreactivity was measured and results are illustrated in **(B–D)**. Note that FosB/ΔFosB levels were significantly increased in CA1 **(B)**, CA3 **(C)**, and DG **(D)** after the first session, in saline-treated mice only (°°*P* < 0.01, °°°*P* < 0.001 vs. controls; Fisher’s least significant difference test). In EP-80317-treated mice, FosB/ΔFosB levels were instead maintained at basal values and were significantly different from saline-treated mice (***P < 0.001 vs. sal-1). Note also that FosB/ΔFosB levels were significantly reduced in the third session of saline-treated mice (^##^*P* < 0.01, ^###^*P* < 0.001 vs. sal-1). Scale bar, 50 μm.

We re-evaluated the effects of repeated exposure to 6-Hz corneal stimulation on FosB/ΔFosB immunoreactivity in the hippocampal regions CA1, CA3, and DG (see Giordano et al., 2015). In these experiments, we quantified the changes occurring after the first and third session of seizure induction. FosB/ΔFosB immunoreactivity was barely detectable in unstimulated control mice (Figure 3A). In all hippocampal regions of saline-treated mice, a prominent increase in FosB/ΔFosB levels was observed after the first 6-Hz corneal stimulation, reaching a statistically significant level in all the considered regions (Figures 3B–D). Interestingly, FosB/ΔFosB immunoreactivity was also significantly higher (*P* < 0.001, Fisher’s LSD test) in saline-treated mice compared to animals treated with EP-80317. On the other hand, treatment with EP-80317 prevented any increase in FosB/ΔFosB levels in the first session of corneal stimulation, in all hippocampal regions. Consistent with previous observations (Giordano et al., 2015), after the third seizure induction FosB/ΔFosB immunoreactivity returned to basal levels in saline-treated mice in all hippocampal regions. Again, no change was noticed in mice treated with EP-80317.

### Levels of p-ERK1/2 Immunoreactivity Progressively Increased in the Hippocampus of Mice Exposed to Repeated 6-Hz Corneal Stimulation

**Figure 4:**
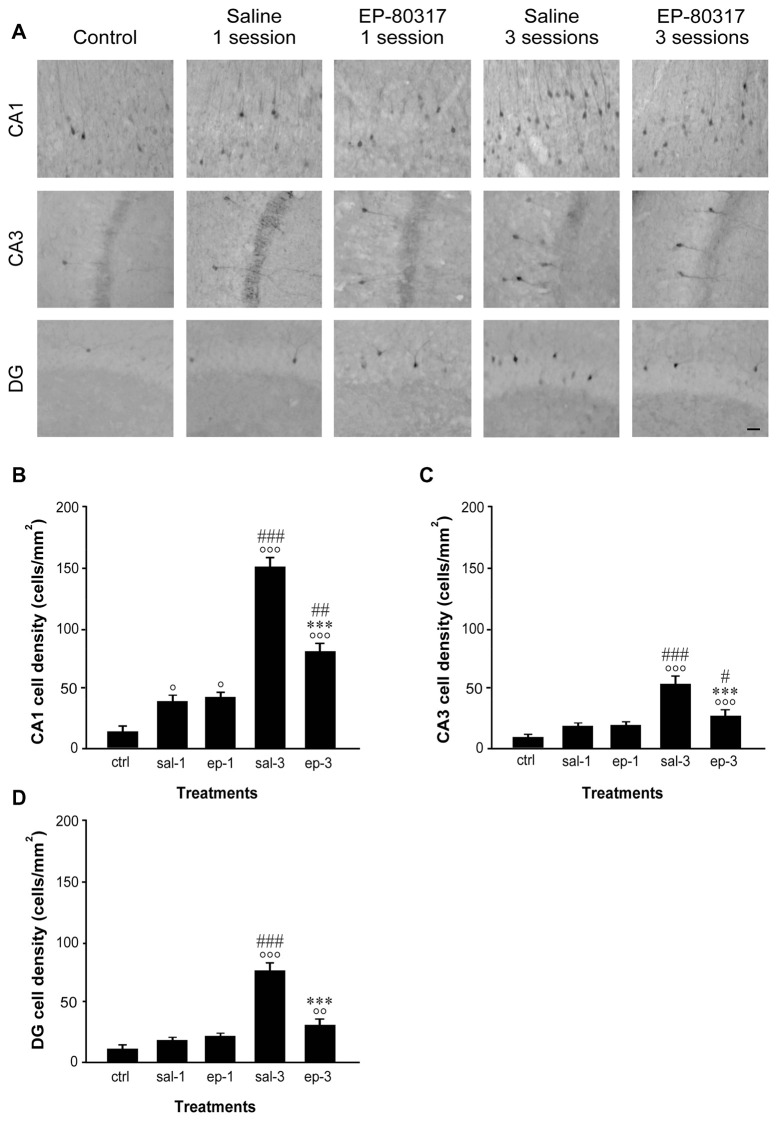
**Changes in phosphorylated extracellular signal-regulated kinases 1/2 (p-ERK1/2) immunoreactivity in hippocampal regions of mice treated with EP-80317 and exposed to different sessions of 6-Hz corneal stimulation.** In **(A)**, p-ERK1/2 immunoreactivity is illustrated in CA1, CA3, and DG, in a representative unstimulated control (ctrl) mouse. Moreover, p-ERK1/2 immunoreactivity is also shown in mice exposed to one or three different sessions of 6-Hz corneal stimulation and pretreated with saline (sal) or EP-80317 (ep). Immunoreactivity was measured and results are illustrated in **(B–D).** Note that p-ERK1/2 expression was initially increased in CA1 (°*P* < 0.05 vs. controls; Fisher’s least significant difference test), both in EP-80317 and saline-treated mice. After the third seizure, immunopositive cells increased also in CA3 **(C)** and DG **(D)** (°°*P* < 0.01, °°°*P* < 0.001 vs. controls) and were higher than in the initial session (^#^*P* < 0.05, ^##^*P* < 0.01, ^###^*P* < 0.001 vs. session 1 of the respectively considered group of treatment). However, the changes observed in mice treated with EP-80317 were less pronounced than in saline-treated mice (***P < 0.001 vs. sal-3). Scale bar, 50 μm.

As found for FosB/ΔFosB, neurons immunopositive to p-ERK1/2 antibodies were scanty in basal condition (Figure 4A). After the first session of seizure induction, p-ERK1/2 immunoreactivity increased significantly in the CA1 hippocampal region of EP-80317 and saline-treated mice (*P* < 0.05 for both groups, Fisher’s LSD test). Notably, after the third session of seizure induction levels of p-ERK1/2 immunoreactivity were markedly elevated in all the analyzed hippocampal regions, with the highest values found in CA1 (Figures 4B–D). EP-80317 administration did not prevent the increase in p-ERK1/2 immunoreactivity observed in the various hippocampal regions, but this change was significantly attenuated (*P* < 0.001 for all hippocampal regions) when comparing these mice with those receiving the saline injection (Figures 4B–D).

### Neurons Immunopositive to p-ERK1/2 Also Expressed FosB/ΔFosB

**Figure 5:**
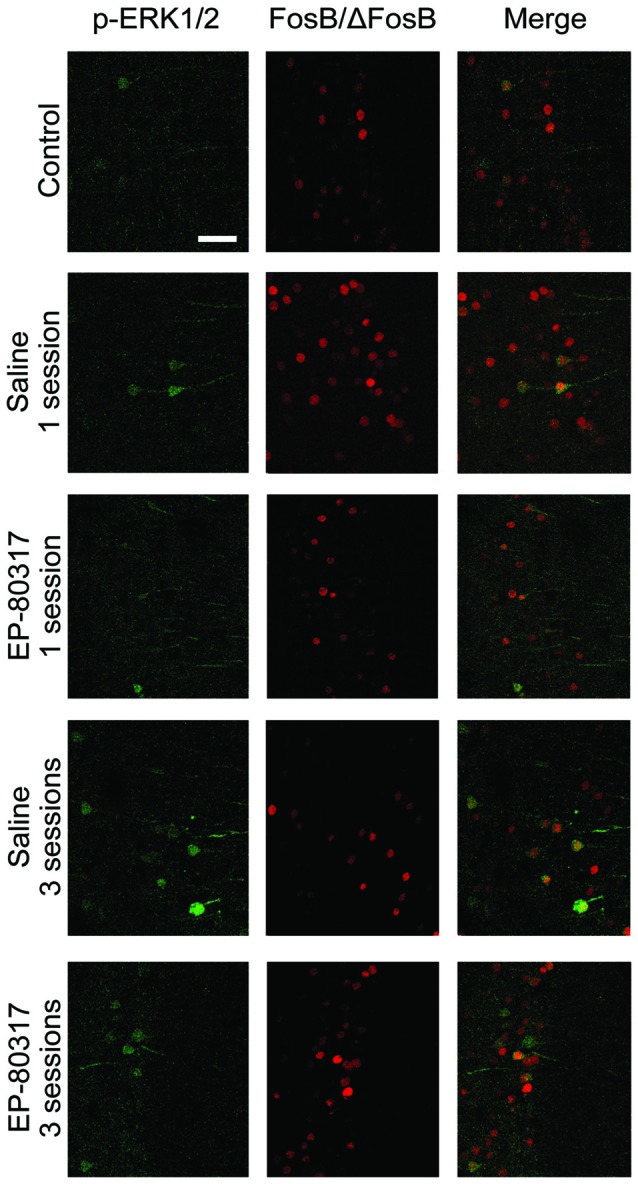
**Double immunofluorescence confocal laser scanning micrographs of mice exposed to repeated 6-Hz corneal stimulation.** Photomicrographs illustrating co-labeling with phosphorylated extracellular signal-regulated kinases 1/2 (p-ERK1/2) (green) and FosB/ΔFosB (red) in CA1 of representative mouse for each group of treatment. Double-label immunofluorescence revealed the coexpression of p-ERK1/2 and FosB/ΔFosB in the experimental groups. Scale bar, 75 μm.

In order to determine whether p-ERK1/2 and FosB/ΔFosB were expressed in distinct cells or coexpressed in the same neurons, we performed a double-immunofluorescence experiment. Cells expressing all the investigated markers (Figure 5) were counted and expressed as percentages of p-ERK1/2 immunopositive neurons. In control animals, an average of 70% ± 23% of p-ERK1/2 immunopositive neurons were also labeled by FosB/ΔFosB antibodies. In treatment groups evaluated after the first session of seizure induction, double-labeled neurons were, respectively, 79% ± 15% in saline-treated mice, and 77% ± 7% in mice treated with EP-80317. After the third seizure induction, double-immunolabeled neurons did not change and were, respectively, 82% ± 4% in saline-treated mice, and 77% ± 7% in mice treated with EP-80317. As no significant differences were found, the increasing number of p-ERK1/2 immunopositive neurons (Figure 4) and, conversely, the reduced immunoreactivity for FosB/ΔFosB (Figure 3), did not affect the overall coexpression of the investigated markers.

## Discussion

We have further explored the repeated 6-Hz corneal stimulation model to characterize a possible marker of the progressive involvement of the hippocampus in seizure worsening, as well as to test the effects of treatment with an anticonvulsant on recurrent seizures and the respective markers of neuronal activation. As major findings of our investigation we determined that: (i) p-ERK1/2 immunoreactivity was progressively induced, especially in CA1, by repeating seizure induction; and (ii) this phenomenon was attenuated by administration of the anticonvulsant EP-80317. Additionally, we confirmed that FosB/ΔFosB immunoreactivity was initially increased, but then it normalized in the course of further seizure induction. This occurred in spite of the consistent seizure aggravation, already noticed in our previous investigation (Giordano et al., 2015).

In our previous experiments, we used an antibody against FosB/ΔFosB chosen on the basis of the transient induction of immunoreactivity observed after exposure to *status epilepticus* (Biagini et al., 2005), as well as for the subsequent reappearance of immunoreactivity for FosB/ΔFosB in epileptic rats (Biagini et al., 2008). Notably, this antibody appeared to be useful in describing the modifications in neuronal network activity even in the presence of different types of seizures (Chen et al., 1995; Curia et al., 2013). However, the ability of this antibody to react with both FosB and ΔFosB precluded the possibility to correctly identify the isoform recognized in our animals. Indeed, the changes observed in corneally stimulated mice were transient, so that the FosB/ΔFosB antibody probably identified the FosB isoform, whose induction is generally followed by the return to basal levels, rather than the ΔFosB variant, which instead accumulates in response to repeated stimulations (Nestler, 2015). Moreover, the finding of increased FosB/ΔFosB immunopositivity after the initial induction, followed by lack of changes in the last seizure, suggests that a desensitization occurred in mice exposed to repeated 6-Hz corneal stimulation. Indeed, this phenomenon of desensitization has been shown for different members of the Fos family in various conditions and also in response to pharmacological treatments, with the notable exception of ΔFosB (Ivkovic et al., 1994; Nakahara et al., 1999; Perrotti et al., 2004; see Nestler, 2015). Precisely, we interpreted the induction of FosB/ΔFosB immunopositivity as due to a novelty effect followed by adaptation, as found also in a different model (Harris et al., 2007).

To address the problem of desensitization encountered with the FosB/ΔFosB antibody, we investigated the pattern of p-ERK1/2 expression in the same condition used for the previous markers (Giordano et al., 2015). Quantification of neurons expressing p-ERK1/2 immunoreactivity revealed a remarkable increase in this marker levels, especially in the CA1 hippocampal region. This region was previously found to present seizure activity only when exposed to repeated 6-Hz corneal stimulations, suggesting the occurrence of an epileptogenic process within the hippocampus (Giordano et al., 2015). It is noteworthy that, in the pilocarpine model of TLE, hippocampal p-ERK1/2 immunoreactivity increased only at the end of the epileptogenic process (Houser et al., 2008). Although the pilocarpine model (Curia et al., 2008) is very different from the repeated 6-Hz corneal stimulation test, it is suggestive that in both conditions there was a coincidence between the increase in p-ERK1/2 immunoreactivity and the appearance of an epileptogenic process. In agreement with this interpretation, it has been reported that phosphorylation of ERK1/2 is stimulated by increased glutamate levels, such as those found in the course of seizures (Jeon et al., 2000; Otani et al., 2003). Moreover, reduction of ERK1/2 phosphorylation was correlated with *in vitro* decreased ictogenesis (Merlo et al., 2004), definitely suggesting a role for p-ERK1/2 in promoting seizures. Consistently, increased p-ERK1/2 levels were shown to be associated with spontaneous epileptic seizures in mice expressing a constitutively active form of MEK1, which activates ERK1/2 (Nateri et al., 2007).

Although the different markers investigated in our experiments followed an opposite trend, we found that the vast majority of neurons expressing p-ERK1/2 were immunopositive for FosB/ΔFosB. A coexpression of these markers was previously demonstrated in about half of neurons immunopositive for p-ERK1/2, in a model of genetic epilepsy (Curia et al., 2013). In normal animals, p-ERK1/2 and FosB/ΔFosB can be both induced by physiological stimuli (Kuroda et al., 2007; Perrotti et al., 2013). Similarly, p-ERK1/2 and FosB/ΔFosB were also upregulated in a model of Parkinson’s disease (Doo et al., 2014). Indeed, lines of evidence support the possibility that p-ERK1/2 can directly regulate FosB levels (Kuroda et al., 2007). Thus, it can be hypothesized that the coexpression of p-ERK1/2 and FosB/ΔFosB occurred in a subpopulation of FosB/ΔFosB immunopositive cells, in which p-ERK1/2 directly stimulated gene expression for FosB. The remaining FosB/ΔFosB immunopositive cells, which did not express p-ERK1/2, were probably activated by different stimuli. We previously hypothesized that the first induction of FosB/ΔFosB immunoreactivity could be due to the stressful procedure required to cause the seizure. Subsequently, animals may become accustomed to the corneal stimulation procedure, so that the effects of stress on FosB/ΔFosB immunoreactivity may subside (Giordano et al., 2015). In this case, neurons expressing both markers could represent a population specifically responsive to seizures, so to be a possible therapeutic target to prevent the epileptogenic changes observed in our model.

The results obtained by treating mice with EP-80317 appear to support this hypothesis. EP-80317 is a ghrelin receptor antagonist and CD36 ligand (Davenport et al., 2005; Callaghan and Furness, 2014) previously found to efficiently counteract seizure induction in rats treated with pilocarpine (Biagini et al., 2011). Here we show for the first time that this anticonvulsant is also able to antagonize seizures induced by 6-Hz corneal stimulation. Especially, EP-80317 was able to prevent the increase in seizure severity, but only temporarily decreased the seizure duration. In both cases, the anticonvulsant effect was associated with attenuation of changes in p-ERK1/2 immunopositivity in the hippocampus, an effect that was more evident in the third session of seizure induction when the increase in p-ERK1/2 immunoreactivity was more significant. This observation confirms that the hippocampus is involved in seizure worsening in the 6-Hz corneal stimulation model. Additionally, the transient effect of EP-80317 on the overall seizure duration suggests the appearance of refractoriness to the tested anticonvulsant. Indeed, the relation with pharmacoresistance is a recognized feature of the 6-Hz corneal stimulation model (Barton et al., 2001; White et al., 2002), and it could be of interest to assess whether the expression of p-ERK1/2 immunoreactivity is associated with refractoriness to AEDs. Definitely, these overall features, including seizure aggravation, refractoriness to anticonvulsants, and the progressive hippocampal involvement, could be part of a process otherwise defined as “secondary epileptogenesis” (Pitkänen and Lukasiuk, 2011; Löscher et al., 2015), which is only partially characterized and that requires the availability of reproducible animal models to be properly addressed.

In conclusion, we provide evidence that some critical processes, such as epileptogenesis and refractoriness to AEDs, could be reproduced especially in the hippocampus exposed to repeated 6-Hz corneal stimulation. Our findings also suggest that ERK1/2 phosphorylation may be involved in both these processes, since levels of p-ERK1/2 increased more dramatically after the third session of corneal stimulation, which was previously demonstrated to precede the appearance of hippocampal seizures (Giordano et al., 2015). Concomitantly, the increase in p-ERK1/2 levels anticipated the reduction in EP-80317 efficacy, suggesting that prevention of ERK1/2 phosphorylation and of the related downstream signals could represent possible novel targets for development of alternative AEDs.

## Author Contributions

GB is responsible for the experimental design, contributed to the acquisition, analysis and interpretation of all the experiments, drafted and revised the work. CG and AMC performed the experiments and contributed to acquisition and analysis of most of the experiments and revised the work. CL and GL performed part of the experiments and revised the work. AT, LB, J-AF and JM provided the anticonvulsant, contributed to data interpretation and revised the work.

## Funding

This study was supported by the Italian Ministry of Health (grant RF-2010-2309921 to GB).

## Conflict of Interest Statement

AT, GB and other inventors share a patent on the possible therapeutic use of growth hormone secretagogues for epileptic disorders (patent 0001399610-2013; http//www.uibm.gov.it/uibm/dati/Avanzata.aspx?load=info_list_uno&id=1800628&table=Invention&#ancoraSearch). The remaining authors have no conflict of interest.

## Footnotes

^1^http://www.mbl.org/atlas232/atlas232_frame.html

^2^http://fiji.sc/Fiji
